# Local Infiltration Analgesia Is Superior to Regional Nerve Blocks for Total Hip Arthroplasty: Less Falls, Better Mobility, and Same-Day Discharge

**DOI:** 10.3390/jcm13164645

**Published:** 2024-08-08

**Authors:** Catalina Baez, Hernan A. Prieto, Abtahi Tishad, Terrie Vasilopoulos, Emilie N. Miley, Justin T. Deen, Chancellor F. Gray, Hari K. Parvataneni, Luis Pulido

**Affiliations:** 1 Department of Orthopaedic Surgery and Sports Medicine, University of Florida, Gainesville, FL 32607, USA; prieth@ortho.ufl.edu (H.A.P.); emilie.miley1@gmail.com (E.N.M.); 2 College of Medicine, University of Florida, Gainesville, FL 32607, USA; tishad100@ufl.edu (A.T.); tvasilopoulos@anest.ufl.edu (T.V.); 3 Florida Orthopaedic Institute, Gainesville, FL 32607, USA; deenju812@gmail.com (J.T.D.); chancellor.gray@gmail.com (C.F.G.); hparvataneni@floridaortho.com (H.K.P.)

**Keywords:** local infiltration analgesia, periarticular injection, peripheral nerve block, regional nerve block, total hip arthroplasty, multimodal pain management, same-day discharge, falls

## Abstract

**Background**: Multimodal analgesia in total hip arthroplasty (THA) provides better pain control, mobility, and reduced side effects compared to monotherapies. Local infiltration analgesia (LIA) and regional nerve blocks (RNBs) are commonly used throughout these protocols. This study aimed to compare these procedures as part of a multimodal analgesia protocol for patients undergoing THA. **Materials and Methods**: A retrospective review of 1100 consecutive elective primary THAs was performed in 996 patients between June 2018 and December 2021. The RNB consisted of a preoperative continuous femoral nerve catheter and single-shot obturator nerve block, and LIA consisted of the intraoperative infiltration of weight-based bupivacaine. **Results**: A total of 579 (52.6%) patients received RNB, and 521 (47.4%) received LIA. Mean oral morphine equivalents (OMEs) during the first four hours postoperatively were significantly lower for LIA group (*p* < 0.001). However, the numeric pain rating scale in the post-anesthesia care unit (PACU) was similar between groups. Patients with LIA had significantly greater first ambulation distance in the PACU (*p* < 0.001), higher successful same-day discharge rate (*p* = 0.029), fewer falls (*p* = 0.041), and less refill OMEs post-discharge (*p* < 0.001) than RNB. **Conclusions**: In the setting of similar pain management between groups and better functional outcomes for LIA, the use of minimally invasive procedures like LIA for pain control following THA is favorable.

## 1. Introduction

The shift towards elective same-day discharge (SDD) in total joint arthroplasty has proven to be safe, satisfactory, and cost-effective. Successful SDD programs highlight pain management strategies catering to early mobility, reduced side effects, and improved surgical workflow and patient experience [1,2,3,4,5,6,7]. Multimodal pain protocols have enhanced postoperative pain control [8,9], leveraging the synergistic effect of different analgesics to provide multifaceted pain management [10,11,12]. These protocols include anesthesia, preemptive analgesia, regional nerve blocks (RNBs), local infiltration analgesia (LIA), cryotherapy, and oral pain medications [8,13,14,15].

Regional nerve blocks consist of the preoperative infiltration of a local anesthetic around a regional nerve, achieving analgesia at the risk of motor blockade [16,17,18,19]. The placement of a catheter allows for the continuous infusion of analgesics into the perineural space which provides prolonged pain control until catheter removal upwards of three to four days later [15,20,21]. Although RNBs have consistently demonstrated to be successful at controlling postoperative pain in total hip arthroplasty (THA) [10,13,16,17], the use for proximal lower limb surgery is sub-optimal as an association has been identified with motor weakness and increased risk of falls in the postoperative period [16,18,22]. An alternative to RNB is LIA, which consists of the systematic intraoperative infiltration of a local anesthetic mixture into the periarticular tissue [14,23]. Frequently, LIA is used in THA and provides adequate pain management and patient satisfaction [15,24,25,26]. However, the effects of LIA are tied to the technique used and the local anesthetic cocktail of choice [23,27].

Local infiltration analgesia and periarticular injections are the most frequent methods for analgesia used in contemporary THA [18,19,20]. The present institution’s standard of care transitioned from RNB to LIA for early postoperative pain management of THA. As such, the aim of this study was to evaluate the impact of this change on pain and functional outcomes in patients undergoing a primary THA. The primary objective was to determine whether pain outcomes (i.e., opioid requirements and pain levels in the post-anesthesia care unit [PACU] and opioids prescribed at discharge) differ between the RNB and LIA intervention. The secondary objective was to determine if functional measures (i.e., first ambulation distance, early falls, and analgesic complications) differ between groups. Finally, the tertiary objective was to compare the rate of successful SDD between groups. The hypotheses were 1.) LIA would manage postoperative pain as effectively as RNB, and 2.) LIA would allow patients to have better mobility and functional outcomes in the immediate postoperative period.

## 2. Materials and Methods

After Institutional Review Board approval was obtained under IRB202200276, a retrospective review was performed on patients who underwent primary THA at a single large tertiary-care academic institution. Patients were included in this study if they underwent a primary THA (i.e., CPT 27130) by one of seven fellowship-trained arthroplasty surgeons between 1 June 2018 and 31 December 2021. Patients were excluded from the study if non-arthroplasty surgeons performed their THA, had a femoral neck fracture, or received other postoperative pain management interventions.

### 2.1. Perioperative Planning and Medication Protocol

All cases received either 1.) intraoperative LIA by the arthroplasty surgeon or 2.) preoperative ultrasound-guided RNB with a continuous femoral nerve block catheter and portable pump for patient-controlled analgesia, paired with a single injection obturator nerve block performed preoperatively by a fellowship-trained regional and acute pain anesthesiologist. Intraoperative LIA consisted of the infiltration of weight-based bupivacaine at 0.25%. The adjusted patient body weight in kilograms was used as a reference for the volume of bupivacaine injected. A continuous femoral nerve block loading dose of 10 to 20 cc of ropivacaine 0.25% was used with a continued infusion rate at 6–8 mL per hour and patient-controlled regional analgesia boluses of 5 cc. The obturator nerve block single shots utilized 10 to 20 cc of ropivacaine at 0.25%. The surgical approach was dependent on surgeon preference and expertise. An anticipated discharge plan (i.e., same-day or inpatient) was a shared decision between the surgeon and the patient, determined by the patient’s physical condition, comorbidities, and social support. The anesthesiology team determined each patient’s type of intraoperative anesthesia (i.e., general or spinal). 

The perioperative protocol remained consistent during this period with exception of the change from RNB to LIA. Medications used as part of multimodal pain management included preoperative dexamethasone 4–8 milligrams (mg) IV immediately before incision for postoperative nausea and single-dose postoperative ketorolac 15 mg IV; while in the PACU, medications varied and were dependent on the patient’s kidney function and associated comorbidities. Postoperatively, oral medications included Tylenol 500 mg every six hours with a maximum 3000 mg per day, celecoxib 100 mg every 12 h for two weeks, and gabapentin 100 mg every night for two weeks for patients under 70 years of age with a low risk of postoperative delirium. 

Additionally, oral opioid medications were prescribed according to a previously defined preoperative opioid stratification protocol; patients were categorized into one of four groups according to self-reported opioid use history: 1.) Opioid Sparing, 2.) Opioid Naïve, 3.) Standard, and 4.) Long-Term Use [28]. Opioid Sparing patients received 21 tablets of Tramadol 50 mg every 6 h as needed, Opioid Naïve patients received 28 tablets of Hydrocodone–Acetaminophen 5–325 mg every four hours as needed, Standard patients received 28 tablets of Oxycodone 5 mg every four hours as needed, and Long-Term Use patients received 21 tablets of Tramadol 50 mg every six hours as needed and 28 tablets of Oxycodone 5 mg every four hours as needed and were instructed to continue the prescribed baseline opioid treatment. 

### 2.2. Outcomes 

Relevant demographic (i.e., age, body mass index [BMI], American Society of Anesthesiologist [ASA] scores, sex, history of anxiety, history of depression, chronic preoperative opioid use, and opioid stratification pathway) and perioperative (e.g., anesthesia type, surgical approach, length of stay [LOS]) data were collected. Of note, patients were classified as “Chronic Opioid Users” when prescribed opioid medication 30 days before surgery. Similarly, classification based on the preoperative opioid stratification protocol categorized patients as “Long-Term Use” [28]. However, discrepancies were noted between these two classifications. The numeric pain rating scale (NRS) was recorded by the nursing and physical therapy staff in the PACU. The NRS is intended to assess the patient’s pain on a scale from zero to ten, where zero represented no pain and ten represented the worst pain imaginable [29,30]. 

Opioids administered were converted into oral morphine equivalents (OMEs) according to the Centers for Disease Control Oral Morphine Milligram Equivalents conversion factors [31], which allows for standardized comparisons between groups. The primary outcome of interest was the total sum of OMEs received in the PACU and the hourly sum of OMEs during the first four hours. Secondary pain outcomes collected in the PACU included the sum of IV-only OMEs, rate of rescue opioids, total and hourly average NRS in the first four hours postoperatively, and rate of rescue nerve blocks administered for breakthrough postoperative pain. Pre- and postoperative opioid prescription and refill data were collected as OMEs. Functional outcomes were first ambulation distance (FAD), postanesthetic complications recorded in the PACU, the rate of early falls (i.e., all falls reported during the first seven days postoperatively), and successful SDD rate (i.e., the rate of patients planned for SDD that were successfully discharged on the same calendar day). 

### 2.3. Statistical Analysis

Statistical analyses were performed using the statistical software IBM SPSS version 28. Categorical measures were summarized with counts and percentages, and continuous measures were summarized using means and standard deviations (SDs). Categorical variables were analyzed for between-group comparisons with chi-square and Fisher’s exact tests. Given the large sample size, parametric tests were used for non-normally distributed single-measurement variables [32]. Single-measurement continuous variables were compared using independent *t*-tests or analysis of variance (ANOVA). Post hoc multivariable analyses were used to control confounders for between-group (i.e., RNB, LIA) differences for the outcomes with significant results. 

Repeated measures were analyzed using generalized linear mixed modeling (GLMM) for a negative binomial distribution with pairwise comparisons and adjusted for multiple comparisons with Sidak correction. Generalized linear mixed modeling is a type of statistical analysis that allows for the development of fixed and random effect regression models that use multilevel continuous and categorical variables regardless of distributions and repeated measures, correcting for non-normality and covariance in the data [32]. Given the non-normal distribution of the repeated measures analyzed and the increased missing data after hour four in the PACU, GLMM was the most appropriate statistical test. In these models, we individually evaluated the fixed effects of time and intervention (RNB vs. LIA) and their interaction on hourly OMEs and the NRS in the PACU. Statistical significance was set to *p* < 0.05.

## 3. Results

A total of 1455 THA cases were identified between 1 June 2018 and 31 December 2021. However, 355 cases were excluded from the study due to the following reasons: 1.) 15 patients had a THA performed by a non-arthroplasty-specific surgeon, 2.) 105 patients had a femoral neck fracture, and 3.) 235 patients received other pain management interventions. The final sample included 1100 THA cases across 996 patients. Of these, 579 cases (52.6%) received RNB and 521 cases (47.4%) received LIA. Demographic characteristics in the population were generally balanced, except for differences between RNB and LIA for sex (*p* = 0.042), ASA scores (*p* < 0.001), depression (*p* = 0.031), and anticipated discharge plan (*p* < 0.001, Table 1).

Several perioperative factors (Table 2) were identified to be significantly different between groups. First, the surgical approach was significantly different (*p* < 0.001), with a larger proportion of posterior approach in the RNB. Similarly, RNB inpatients had significantly more extended stays in the PACU than LIA inpatients (*p* < 0.001). However, differences in type of anesthesia, surgical time, LOS, and duration in the PACU for SDD patients were not significantly different between groups. 

As differences were identified among groups at baseline (i.e., sex, ASA score, depression, and anticipated discharge plan), we performed multivariable analyses to control for baseline differences. No confounding effects were identified between the covariates and the main effect of the interventions (i.e., RNB and LIA) with *p*-values ranging from 0.125 to 0.971.

### 3.1. Pain Outcomes 

#### 3.1.1. Opioid Requirements

**Figure 1:**
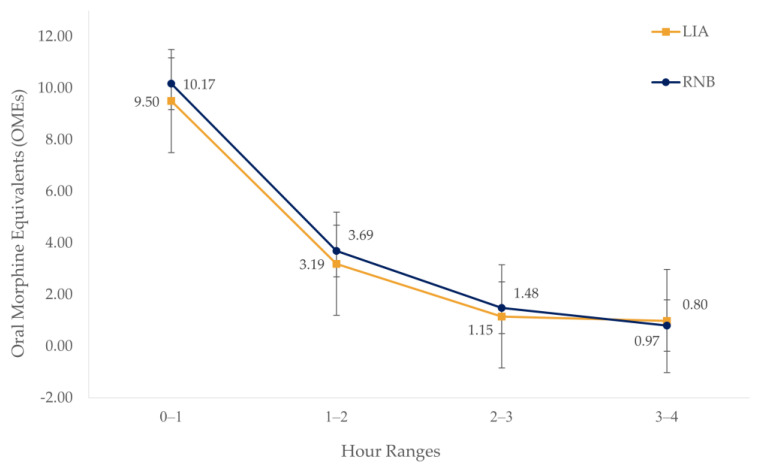
Mean OMEs received in PACU.

Mean OME requirements during the first four hours in the PACU were significantly different between groups (F[1, 2220] = 11.51, *p* < 0.001; Figure 1), where the LIA group averaged less OMEs (3.8 ± 6.6) than the RNB group (4.5 ± 7.3; *p* < 0.001). Additionally, OME requirements significantly decreased over time regardless of the group (F[3, 2220] = 74.44, *p* < 0.001). Although the LIA group required significantly fewer OMEs (9.5 ±8.5) than RNB group (10.2 ± 9.1; *p* = 0.002) at hours one and three (LIA = 1.2 ± 3.7, RNB = 1.5 ± 4.1; *p* = 0.020; Figure 1), the combined effect of the groups across time on OMEs in the PACU was not statistically significant (F[3, 2220] = 0.83, *p* = 0.478).

The sum of intravenous opioids required in the PACU was not significantly different (t[1093.52] = 1.47, *p* = 0.143). However, patients in the LIA group required almost double as many OMEs as the patients in the RNB group (Table 3). In addition, there were significantly more patients in the LIA group (81.2%) that required rescue opioids in the PACU compared to the RNB group (74.1%; χ^2^ = 7.91, *p* = 0.006, Table 3). Patients in the LIA group had 1.5 higher odds (95% CI [1.1, 2.0]) of needing rescue opioids during their initial PACU stay than RNB patients. However, the mean sum of all OMEs received during their PACU stay was not significantly different (t[1094.67] = 0.24, *p* = 0.809) between the LIA (12.8 ± 10.8) and RNB (12.6 ± 12.6) groups.

The rate of rescue nerve blocks required in the PACU (n = 20) was twice as high in the RNB group (2.4%, n = 14) than in the LIA group (1.2%, n = 6, Table 3), though this difference was not statistically significant (χ^2^ = 2.46, *p* = 0.174). The most frequently performed rescue nerve block was a single-shot lateral femoral cutaneous nerve block (35%, n = 7), followed by combination blocks with more than one targeted nerve (30%, n = 6). The remaining 35% (n = 7) of the rescue blocks performed were one single-shot pectineus nerve block, one single-shot sciatic nerve block, two pericapsular nerve group blocks, two field blocks, and one epidural block.

Lastly, the mean OME refills were significantly different between groups (t[195] = −4.5, *p* < 0.001), with patients in the RNB group receiving more opioids (282.2 ± 182.1) than LIA group (176.7 ± 126.9). However, OMEs prescribed preoperatively for post-discharge pain were not significantly different between groups (t[998.80] = 0.002, *p* = 0.999), with patients in the LIA group having a mean of 396.2 ± 711.9 OMEs for the first 90 days (i.e., average of 4.4 ± 7.9 per day) and the RNB group having a mean of 396.2 ± 574.0 OMEs for the first 90 days (i.e., average of 4.4 ± 6.4 per day).

#### 3.1.2. Numeric Pain Rating Scale

**Figure 2:**
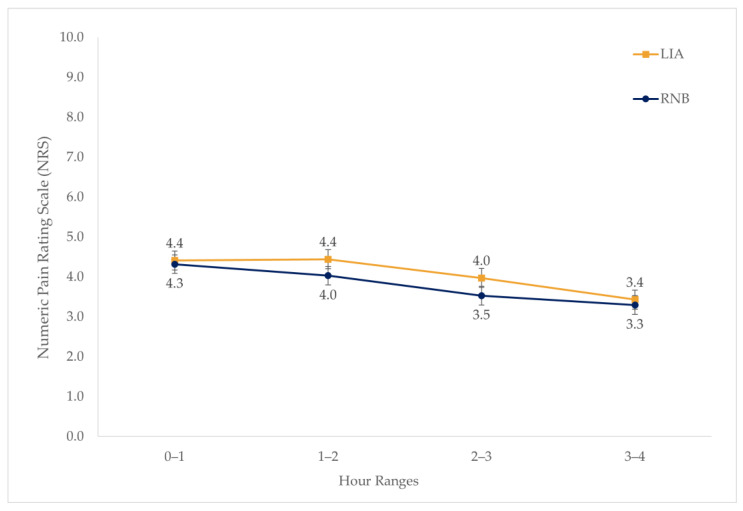
Mean hourly NRS recorded in PACU.

The average NRS recorded for patients in the PACU did not significantly differ between groups (t[1093.52] = 1.66, *p* = 0.097), with patients in the LIA group having a mean score of 4.2 ± 2.4 compared to a mean score of 4.0 ±2.6 in the RNB group. Additionally, the mean NRS recorded during the first four hours in the PACU was not significantly different for either group (F[1, 3459] = 3.07, *p* = 0.080). Pain levels reported in the PACU significantly decreased over time (F[3, 3459] = 23.37, *p* < 0.001). The combined effect of the group and time on the mean hourly NRS in the PACU showed an overall significant difference (F[3, 3459] = 3.08, *p* = 0.027), specifically at hour two (LIA = 4.4 ± 2.7, RNB = 4.0 ± 3.0; *p* = 0.014) and hour three (LIA = 4.0 ± 2.5, RNB = 3.5 ± 2.8; *p* = 0.014; Figure 2).

### 3.2. Functional Outcomes

The first ambulation distance in the PACU was significantly greater (t[611.85] = 5.10, *p* < 0.001) for patients in the LIA group (41.7 ft ± 68.5 ft) than RNB group (22.2 ft ± 38.3 ft). Notably, 40 patients in the RNB group could not ambulate in the PACU due to quadriceps weakness, while only three patients in the LIA group encountered a similar situation. Patients who received an RNB reported significantly (*p* = 0.041) more falls (1.7%, n = 10) compared to those who received LIA (0.4%, n = 2). This translated to 4.6 higher odds (95% CIs: 1.0–20.9) of falling during the first seven days after THA for patients managed with RNB over those with LIA. Postanesthetic complications recorded in the PACU were not significantly different between groups (χ^2^ = 0.01, *p* = 0.918), with 6.6% reported for the LIA group, and 6.7% reported for the RNB group. Finally, the rate of successful SDD was significantly greater (χ^2^ = 4.79, *p* = 0.029) in LIA (90.1%) than in RNB (80.6%). Thus, of those patients who were anticipated to have an SDD, those who received LIA had 2.2 higher odds (95% CIs: 1.07–4.5) of being successfully discharged on the same day than patients who received RNB.

## 4. Discussion

Increasing demand for adequate postoperative pain management that avoids motor blockade and allows for early patient mobilization has led to the quick adoption of LIA for immediate postoperative pain management in patients undergoing THA. Although multiple studies have compared the efficacy of LIA and different regional nerve blocks on postoperative pain after THA [14,15,22,24,25,26,33,34], only few studies have compared the effects of these interventions during the immediate postoperative period [14,25,26]. Additionally, modern SDD Enhanced Recovery After Surgery (ERAS) protocols must factor in baseline patient function, safety, and pain control, as most patients are now out of the hospital setting during early recovery. Consequently, our study aimed to determine if there was a difference in pain and functional outcomes between LIA and RNB for THA during the patient’s stay in the PACU.

Patients consumed less OMEs across time spent in the PACU which suggests that opioid requirements decreased significantly during the first four hours after surgery regardless of the intervention groups. Fewer patients in the RNB group required opioids in the PACU than patients in the LIA group, which demonstrates 1.5 higher odds of requiring rescue opioids during this time. However, the sum of OMEs received remained the same between groups. Patients in the RNB group received double the rate of rescue nerve blocks than LIA patients, although this difference was not statistically significant. Furthermore, RNB patients had access to patient-controlled analgesia via the continuous femoral nerve block, introducing a pain-controlling pathway that LIA patients lacked. As such, the clinical effect of LIA and RNB on opioid requirements in the immediate postoperative period was similar, underscoring the consideration of each patient’s functional goals in deciding between these two interventions [24].

Preoperatively prescribed OMEs for postoperative pain did not differ between groups, reflecting the arthroplasty division’s efforts to standardize opioid prescription in compliance with state and federal laws [28]. Demographic data obtained demonstrated a balance across opioid stratification of patients, and unsurprisingly, records of prescribed OMEs were similar between groups. The OME conversions for opioid refills given during the first 90 days after surgery work as a surrogate for pain management beyond hospitalization. Continuous RNB patients consumed greater amounts of opioids during this period, contradicting the theoretical concept of prolonged analgesia for RNB [20,21,22].

The average NRS in the PACU was similar between groups, but decreased significantly across time achieving a minimal clinically important difference of one point between hours one and four [29,30]. Whether this is due to the interventions under analysis, the consumed opioids in the PACU, or the natural history of pain in the postoperative period is unclear from this dataset. However, findings from this study are comparable to other authors who have identified similar pain control between LIA and RNB groups [25,35,36,37]. Postanesthetic complications did not differ between groups which may reflect the balance in types of anesthesia used and opioids consumed across groups, which other authors have also identified similar findings [14,15,22,25,26,37]. Given that RNB and LIA provide similar levels of pain control, functional outcomes may have a greater weight in directing pain management. First, ambulation distance reflects adequate pain control and directly influences early discharge in total joint arthroplasty [3]. Within this study sample, LIA patients ambulated an average of 20 ft more than RNB patients in the PACU, with more RNB patients reporting quadricep weakness as the cause of ambulation failure. Similarly, falls soon after surgery are an essential indicator of functional capacity after THA. Patients in this study who received an RNB had 4.6 higher odds of having a fall within the first week after surgery than those who received LIA. Two of the RNB patients who fell associated the event with quadriceps weakness. On the contrary, LIA patients only reported two falls due to slip and fall. These findings are supported by a previous systematic review and meta-analysis which identified continuous lumbar plexus block associated with an increased risk of falls compared to single-shot lumbar plexus block, wherein this type of block also targets the femoral and obturator nerves used by our team [18]. Furthermore, the American Association of Hip and Knee Surgeons, the American Academy of Orthopedic Surgery, the American Society of Regional Anesthesia and Pain Management, the Hip Society, and the Knee Society combined safety and efficacy panel has recently advised against continuous and single-shot femoral nerve blocks due to their increased risk of motor weakness [22].

Finally, the successful SDD rate was significantly greater in patients who obtained a LIA than RNB, where the LIA group had 2.2 higher odds of achieving SDD. For SDD to be completed, patients had to ambulate safely, have well-controlled pain, no vomiting or nausea, and have normal bladder function. Since pain levels and complication rates are not different between interventions, the primary catalyst for successful SDD may be related to factors that allow for improved ambulation in the PACU. A previous study in the present institution found that longer first ambulation distances were associated with successful SDD [3], further supporting pain management techniques that spare muscle function, such as LIA.

This study is not without its limitations. First, as this study was retrospective in nature, inherent difficulties existed in data collection and analysis. However, the statistical analyses used attempted to consider these structural limitations and control for them. Second, there were baseline differences between groups in terms of sex, ASA score, depression rates, anticipated discharge plan, and surgical approach. However, post-hoc multivariable regression was performed to control for these differences and assess the confounding effect on the significant outcomes. No variables were identified to significantly confound the expected impact of the intervention on the outcomes. However, this study may have been underpowered to address these confounding effects, and the results should be interpreted considering these differences; as female sex, depression diagnoses, and surgical approach have been conflictingly associated with higher levels of postoperative pain [38,39,40,41,42,43]. Regardless, these data demonstrated similar pain levels and opioid requirements between groups.

Third, although not statistically significant, there is a general trend toward increased use of spinal anesthesia over general anesthesia, potentially introducing bias to the data. The dose of spinal anesthesia and the adjuvants used has been reported to significantly affect pain management, LOS, and complications after hip surgery which could confound the effects of RNB and LIA on pain and functional outcomes [8,44,45,46,47]. Nonetheless, the protocols for ERAS favor using spinal anesthesia for SDD management [48]. Ultimately, these differences noted between groups reflect changes adopted over time by the anesthesia and arthroplasty departments for preferred patient management practices. Fourth, the transition to LIA was shortly followed by the 2020 coronavirus pandemic and Medicare’s exclusion of THA from the inpatient-only list. This led to an increased volume of SDD THAs, echoed by the significant differences amongst groups for anticipated discharge plans. However, these changes did not affect the pain intervention received by our patients nor the calculation for successful SDD. Fifth, although the use of a stratification pathway for opioid prescription is an excellent way to curve physician opioid over-prescribing, the existence of this pathway limits the analyzability of our data for preoperatively prescribed OMEs. It is unlikely that these were related to the pain intervention given that the prescription pathway accounts for expected opioid needs at home regardless of the planned perioperative pain management strategy [28]. Data on refills better represent the effect of OMEs required postoperatively. Lastly, this was a single-institution series, potentially limiting the generalizability of the data. This was partially compensated for by the inclusion of THAs performed by seven different fellowship-trained orthopedic surgeons, increasing the generalizability of the results. Nonetheless, this study provides the largest sample for comparison to date.

## 5. Conclusions

Data comparing LIA and RNB interventions as immediate postoperative pain management methods demonstrated minimal differences in objective and subjective pain measures. Additionally, this study provided further evidence that postoperative pain after THA is adequately controlled with less invasive procedures such as LIA. Functional outcomes analyzed in this study support using LIA over RNB as patients who received this treatment had increased first ambulation distance, greater odds of a successful SDD, fewer postoperative falls, and similar pain outcomes to RNB. As such, these findings further support using LIA for primary elective THA.

## Figures and Tables

**Table 1:** Patient demographics.

	Total N = 1100	RNB n = 579	LIA n = 521	*p*-Value *
Age, mean (SD)	65 (11.1)	64 (11)	65 (11)	0.185
BMI, mean (SD)	31 (7.5)	31 (7)	31 (8)	0.413
Sex, n (%)				**0.042**
Male	482 (43.8)	237 (40.9)	245 (47.0)	
Female	618 (56.2)	342 (59.1)	276 (53.0)	
ASA Score, n (%)				**<0.001**
1	4 (0.4)	2 (0.3)	2 (0.4)	
2	360 (32.7)	157 (27.1)	203 (39.0)	
3	712 (64.8)	412 (71.2)	300 (57.6)	
4	24 (2.2)	8 (1.4)	16 (3.1)	
Anxiety Diagnosis, n (%)	405 (36.8)	215 (37.1)	190 (36.5)	0.819
Depression Diagnosis, n (%)	328 (29.8)	189 (32.6)	139 (26.7)	**0.031**
Preoperative Opioid Stratification, n (%)				0.542
Opioid Sparing	52 (4.7)	31 (5.4)	21 (4.0)	
Narcotic Naïve	803 (73.0)	411 (71.0)	392 (75.2)	
Standard	120 (10.9)	68 (11.7)	52 (10.0)	
Long-Term Use	100 (9.1)	54 (9.3)	46 (8.8)	
Chronic Opioid User, n (%)	181 (16.5)	86 (14.9)	95 (18.2)	0.131
Anticipated Discharge Plan, n (%)				**<0.001**
Inpatient	745 (68.0)	508 (88.3)	237 (45.5)	
Same-day discharge	351 (32.0)	67 (11.7)	284 (54.5)	

Abbreviations: RNB: regional nerve block, LIA: local infiltration analgesia, BMI: body mass index, ASA Score: American Society of Anesthesiologists Physical Status Classification. * All bolded *p*-values indicate a statistically significant difference at *p* < 0.05.

**Table 2:** Perioperative outcomes.

	Total N = 1100	RNB n = 579	LIA n = 521	*p*-Value *
Type of Intraoperative Anesthesia, n (%)				0.096
General	854 (77.6)	461 (79.6)	393 (75.4)	
Spinal	246 (22.4)	118 (20.4)	128 (24.6)	
Surgical Approach, n (%)				**<0.001**
Anterolateral	14 (1.3)	13 (2.2)	1 (0.0)	
Direct anterior	319 (29.0)	139 (24.0)	180 (34.5)	
Posterior	767 (69.7)	427 (73.7)	340 (65.3)	
Duration of surgery (mins), mean (SD)				
Same-day discharge	87.0 (20.0)	89 (20.0)	86 (20.0)	0.832
Inpatient	98.4 (29.4)	95 (28.0)	98 (29.0)	0.192
Time in PACU (hours), mean (SD)				
Same-day discharge	5.0 (1.4)	5.2 (1.4)	4.9 (1.4)	0.930
Inpatient	4.5 (3.4)	4.6 (3.0)	4.5 (3.3)	**<0.001**
Length of stay (hours), mean (SD)				
Same-day discharge	9.3 (1.9)	9.5 (2.2)	9.28 (1.8)	1.000
Inpatient	55.9 (37.2)	43.9 (38.0)	52.24 (37.8)	0.625

Abbreviations: RNB: regional nerve block, LIA: local infiltration analgesia, PACU: post-anesthesia care unit. * All bolded *p*-values indicate a statistically significant difference at *p* < 0.05.

**Table 3:** Pain and functional outcomes.

	Total N = 1100	RNB N = 579	LIA N = 521	*p*-Value *
Pain Outcomes				
Rate of opioids received in PACU, (%)	852 (77.5)	429 (74.1)	423 (81.2)	**0.005**
Total OMEs in PACU, mean (SD)	12.7 (11.8)	12.6 (12.6)	12.8 (10.7)	0.809
Rescue IV OMEs in PACU, mean (SD)	9.2 (51.0)	6.9 (14.2)	11.7 (72.5)	0.143
Rate of rescue nerve blocks in PACU, n (%)	20 (1.8)	14 (2.4)	6 (1.2)	0.117
Prescribed OMEs, mean (SD)				0.938
Total	395.9 (642.7)	396.2 (574.0)	396.2 (711.9)	
Daily	4.4 (7.1)	4.4 (6.0)	4.4 (7.9)	
Postoperative opioid refills (90 d), mean (SD)				
Number of refills	1.4 (0.8)	1.4 (0.7)	1.4 (0.9)	0.716
Total refill OMEs	221.1 (160.9)	282.2 (182.1)	176.7 (126.9)	**<0.001**
Daily refill OMEs	2.5 (1.7)	3.1 (2.0)	1.96 (1.4)	**<0.001**
Functional outcomes				
Postanesthetic complications, n (%)	73 (6.6)	38 (6.6)	35 (6.7)	0.918
FAD in PACU (ft), mean (SD)	31.2 (55.1)	22 (37.0)	42 (68.0)	**<0.001**
Successful same-day discharge rate, n (%)	310/351 (88.3)	54/67 (80.6)	256/284 (90.1)	**0.029**
Early falls, n (%)	12 (1.1)	10 (1.7)	2 (0.4)	**0.041**

Abbreviations: RNB: regional nerve block, LIA: local infiltration analgesia, PACU: post-anesthesia care unit, OMEs: oral morphine equivalents, IV: intravenous, FAD: first ambulation distance. * All bolded *p*-values indicate a statistically significant difference.

## Data Availability

The dataset analyzed during the study is not publicly available per the study protocol. De-identified data may be available from the corresponding author with permission from the University of Florida upon reasonable request.
